# Quality Indicators and Clinical Outcomes of Acute Stroke: Results from a Prospective Multicenter Registry in Greece (SUN4P)

**DOI:** 10.3390/jcm13030917

**Published:** 2024-02-05

**Authors:** Eleni Korompoki, George Ntaios, Argyro Tountopoulou, Georgios Mavraganis, Evangelos Tsampalas, Ioannis Kalliontzakis, Sofia Vassilopoulou, Efstathios Manios, Christos Savopoulos, Haralampos Milionis, Athanasios Protogerou, Nikolaos Kakaletsis, Petros Galanis, Daphne Kaitelidou, Olga Siskou, Konstantinos Vemmos, on behalf of the SUN4P Project Collaborators

**Affiliations:** 1 Department of Clinical Therapeutics, National and Kapodistrian University of Athens, 11528 Athens, Greece; giomavraganis@gmail.com (G.M.); stathismanios@yahoo.gr (E.M.); 2 Department of Internal Medicine, Faculty of Medicine, School of Health Sciences, University of Thessaly, 41334 Larissa, Greece; gntaios@med.uth.gr; 3 1st Department of Neurology, Eginition Hospital, National and Kapodistrian University of Athens, 11528 Athens, Greece; atounto@yahoo.gr (A.T.); svasilop@med.uoa.gr (S.V.); 4 Department of Medicine, Arcadia General Hospital, 22100 Tripoli, Greece; tsampalas_e65@yahoo.gr; 5 Department of Neurology, General Hospital of Chania, 73300 Chania, Greece; jkaliontzis@hotmail.com; 6 First Propaedeutic Internal Medicine Department, Aristotle University of Thessaloniki, AHEPA Hospital, 54636 Thessaloniki, Greece; chrisavopoulos@gmail.com; 7 Department of Internal Medicine, School of Medicine, University of Ioannina, 45500 Ioannina, Greece; hmilioni@uoi.gr; 8 Clinic-Laboratory of Pathophysiology, First Department of Propeadeutic Internal Medicine, Laiko Hospital, Medical School, National and Kapodistrian University of Athens, 11527 Athens, Greece; aprotog@med.uoa.gr; 9 Second Propedeutic Department of Internal Medicine, Aristotle University of Thessaloniki, Hippokrateion General Hospital of Thessaloniki, 54643 Thessaloniki, Greece; kakaletsisnikos@yahoo.gr; 10 Center for Health Services Management and Evaluation, Nursing Department, National and Kapodistrian University of Athens, 11527 Athens, Greece; pegalan@nurs.uoa.gr (P.G.); dkaitelid@nurs.uoa.gr (D.K.); olsiskou@nurs.uoa.gr (O.S.); 11 Department of Tourism Studies, University of Piraeus, 18534 Piraeus, Greece; 12 Hellenic Cardiovascular Research Society, 11528 Athens, Greece; vemmosk@gmail.com

**Keywords:** stroke, quality, registry, thrombolysis, Greece

## Abstract

Aim: The Stroke Units Necessity for Patients (SUN4P) project aims to provide essential data on stroke healthcare in Greece. Herein, we present results on established quality indicators and outcomes after first-ever stroke occurrences. Methods: This prospective multicenter study included consecutive patients admitted to nine hospitals across Greece in 2019–2021. Descriptive statistics were used to present patients’ characteristics, key performance measures and stroke outcomes. Results: Among 892 patients, 755 had ischemic stroke (IS) (mean age 75.6 ± 13.6, 48.7% males) and 137 had hemorrhagic stroke (HS) (mean age 75.8 ± 13.2, 57.7% males). Of those, 15.4% of IS and 8% of HS patients were treated in the acute stroke unit (ASU) and 20.7% and 33.8% were admitted to the intensive care unit (ICU) or high-dependency unit (HDU), respectively. A total of 35 (4.6%) out of 125 eligible patients received intravenous alteplase with a door-to needle time of 60 min (21–90). The time to first scan for IS patients was 60 min (31–105) with 53.2% undergoing a CT scan within 60 min post presentation. Furthermore, 94.4% were discharged on antiplatelets, 69.8% on lipid-lowering therapy and 61.6% on antihypertensives. Oral anticoagulants (OAC) were initiated in 73.2% of the 153 IS patients with atrial fibrillation (AF). Among the 687 IS patients who survived, 85.4% were discharged home, 12% were transferred to rehabilitation centers, 1.2% to nursing homes and 1.3% to another hospital. Conclusions: The SUN4P Registry is the first study to provide data from a prospectively collected cohort of consecutive patients from nine representative national hospitals. It represents an important step in the evaluation and improvement of the quality of acute stroke care in Greece.

## 1. Introduction

Stroke remains the second leading cause of death and the third cause of disability-adjusted life years worldwide in 2019 [1]. While the age-standardized stroke incidence rates are decreasing globally, the absolute numbers of stroke incidence, prevalence and mortality are on the rise. Consequently, stroke poses a significant global public health challenge with devastating consequences for families, communities and healthcare systems.

The burden of stroke in the last decade in Greece fluctuates widely across different regions with an annual stroke incidence ranging from 117 to 534 cases per 100,000 inhabitants [2,3,4]. Despite almost 35,000 patients suffering and 16,000 dying from stroke annually in Greece [1,5], the management in the acute phase of stroke remains heterogeneous. Limited resources, the absence of implementation systems, the lack of national guidelines, and the absence of a comprehensive national strategy are among the factors contributing to the gap between evidence-based medicine and clinical practice. According to a survey conducted by the European Stroke Organization, stroke care in Greece ranks lowest in terms of the admission of patients to dedicated stroke units and access to reperfusion treatments [6]. However, data deriving directly from national stroke registries for the holistic stroke management in Greece are lacking. National health data from well-designed clinical registries play an important role in evaluating healthcare delivery and supporting quality improvement for stroke patients [7] and provide real-world data of clinical practice contributing to quality improvement and education [8].

The SUN4P (Stroke Unit Necessity for Patients) project represents a multicenter prospective registry collecting data from nine hospitals across Greece. The project aims to support the efforts of various factors (e.g., professionals, policy makers, patients), to improve clinical outcomes for stroke patients and enhance the sustainability of the healthcare system by minimizing waste of resources. In this study we present essential data on pre-hospital features, acute stroke management quality indicators and in-hospital primary and secondary performance measures in relation to hard clinical outcomes at 3 months of follow up.

## 2. Materials and Methods

### 2.1. Study Population Selection and Data Collection

This is a prospective cohort multicenter study of patients with first-ever acute stroke, including hemorrhagic and ischemic stroke, (ICD-10 codes: I61, I63 and I64) admitted within 48 h of the onset of symptoms to nine National Health System (NHS) or University hospitals across Greece (Supplementary Figure S1). Patients with previous stroke incidences have been excluded from the study. Participating hospitals have been selected as representative of the country’s demographics and geography. All patients were followed up for three months. The study was carried out from September 2019 to September 2021. The study protocol was approved by the Bioethics Committee of the National and Kapodistrian University of Athens (NKUA) and the Ethical Boards and Scientific Committees of the contributing hospitals. All registered patients or their representatives gave written informed consent before entering the study. The study was registered in the Clinical Trials Registry (NCT04109612, date of approval 27 September 2019).

### 2.2. Study Design

A multi-variable stroke registry was developed based on the recommendations of the European Stroke Organization, the American Heart Association and the experiences of other hospital based registries.

An expert panel was asked to provide input on variable selection and definitions. Data collection instruments and variable definitions within SUN4P were aligned with available databases in acute stroke care (Athens Stroke Registry [9], Acute Stroke Registry and Analysis of Lausanne [10]). A predefined standardized questionnaire based on an extensive manual was used for prospective data collection. Data at each center were collected by at least one trained physician or stroke nurse. Data entry and monitoring were performed using a flow chart web-based multichannel platform. The SUN4P platform incorporated the whole business logic and validation controls of electronic Case Report Forms (eCRF). It supported innovative and multichannel data collection methods, provided visual exploration of the major quality indicators and supported effective scheduling and management of the follow-up monitoring of patients.

### 2.3. Data Recording

The registry incorporated demographic data, stroke characteristics and quality indicators at 3 phases (Supplementary Figure S2). First, at the point of admission: baseline demographic characteristics, prehospital and in-hospital delays, major stroke subtypes, medical history and risk factors and neurological impairment were assessed by the National Institutes of Health Stroke Scale (NIHSS) [11]. Second, during inpatient care: reperfusion treatment (thrombolysis and thrombectomy), stroke work-up, medical treatment, management of complications, early rehabilitation and discharge destination. Third, at 3 months post stroke, outcomes based on modified Rankin Scale (mortality and disability) [12].

### 2.4. Quality Indicators Selection

Variables that serve as quality indicators at the acute stroke setting have been selected based on an extensive literature review incorporating international guidelines and good clinical practice strategies [13,14,15]. Additional data regarding pre-hospital management and in-hospital work-up that may serve as secondary quality indicators have been also collected. For each indicator, the number of patients who actually received an intervention/investigation was divided by all potentially eligible patients.

### 2.5. Statistical Analysis

Data are analyzed and presented by major stroke subtype. Continuous variables are presented as mean (±standard deviation, SD) or median values (Inter quartile range, IQR) and categorical covariates as absolute numbers and proportion (%). The student’s *t*-test was applied for continuous variables; the chi-square or Fisher’s exact test was applied for categorical variables. The Kolmogorov–Smirnov test and normal Q-Q plots were used to test the normality of the continuous variables. For patient outcomes, the modified Rankin Scale (mRS) was used. Statistical analysis was performed with the Statistical Package for Social Sciences software (IBM Corp. Released 2012. IBM SPSS Statistics for Windows, Version 21.0. Armonk, NY, USA: IBM Corp.).

## 3. Results

**Figure 1:**
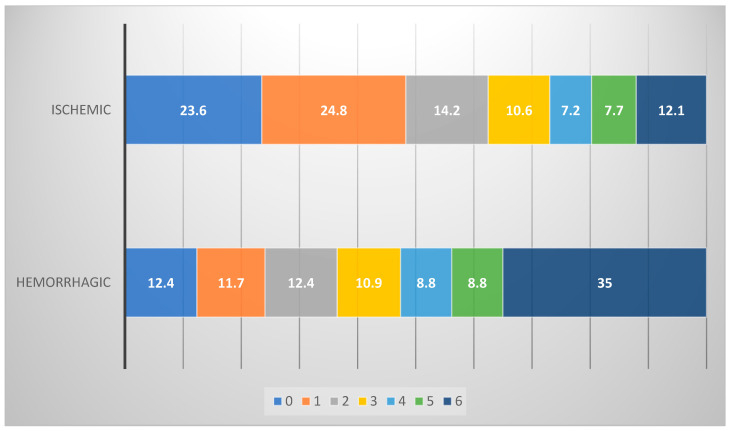
Stroke outcomes at 3 months stratified by major stroke subtype (%) based on modified Rankin Scale (0–6).

During the study period, 913 consecutive patients with first-ever stroke were registered in the participating hospitals, and 892 completed the follow-up. There were 755 (84.6%) cases with ischemic stroke (IS) (mean age 75.6 ± 13.6, 48.7% males) and 137 (15.4%) with hemorrhagic stroke (HS) (mean age 75.8 ± 13.2, 57.7% males) (Table 1). Stroke severity on admission was significantly higher in patients with HS compared to IS [median NIHSS score 12 (5–23) vs. 6 (3–11), *p* = 0.001] (Table 1), resulting in significantly greater in-hospital and 90-day mortality (45% vs. 12.2%, *p* < 0.001), respectively. HS patients had a higher likelihood to be dependent ompared to those with IS (40.1% vs. 34% and 28.5% vs. 25.3%, respectively) (Table 2, Figure 1). Patients with IS and HS shared similar stroke risk factors and comorbidities except the history of diabetes mellitus which was more common among IS patients, 27.5% vs. 16.1%, *p* = 0.004 (Table 1).

### 3.1. Quality Indicators

At the acute phase of stroke, only 15.4% of IS patients were treated in an acute stroke unit (ASU) and 20.7% had been admitted to an intensive care unit (ICU) or high-dependency unit (HDU). In the case of HS, 8% of patients were admitted to ASU and 33.8% to ICU/HDU (Table 2). During their hospitalization, 94.3% for IS and 95.6% for HS patients had a CT scan within the first 24 h and 53.4% of IS had vascular imaging (Table 2). Time to first scan for IS patients was 60 min (21–105) and 53.2% had a CT scan within 60 min post admission. With regards to acute reperfusion treatment, among 125 potentially eligible patients (16.6%), 35 (4.6%) received intravenous alteplase. Door-to-needle time was 60 min (21–90) (Table 2). With regard to the prevention of stroke-related complications, screening for dysphagia was performed in 21.6% of IS patients and in 41.6% of HS patients (*p* < 0.001). Low molecular weighted heparin (LMWH) for venous thromboembolism prophylaxis were administered in 65% of IS patients and in 39.4% of HS patients (*p* < 0.001) (Table 2). In-hospital assessment by physiotherapist/occupational therapist was performed in almost one third of stroke patients (29.8% of IS and 33.6% of HS) (Table 2). Secondary prevention treatment was started in the vast majority of IS patients: 94.4% without indication for OAC were discharged on antiplatelets and 73.2% of patients with AF were discharged on OAC. Of the 687 IS survivors, 69.8% were discharged on lipid lowering therapy and 61.6% on antihypertensive therapy. Active smokers received smoking cessation advice in 78% of cases (Table 2). Carotid endarterectomy within 14 days post symptom onset was performed in 6 out of 26 eligible patients (23.1%) (Table 2). Most of the stroke survivors, 85.4% of IS and 65% of HS, were discharged to their homes.

### 3.2. Secondary Performance Measures

All patients had at least one CT scan during their hospital stay. Patients with HS had a second CT scan more often than those with IS (38% vs. 23.4%, *p* = 0.001) whereas on the contrary, magnetic resonance imaging (MRI) was performed more often in patients with IS than HS (36.8% vs. 22.6%, *p* = 0.001). Almost one third of IS patients underwent cardiac evaluation with arrhythmia detection (30.5%) (Table 2), 39.5% were assessed with transthoracic echocardiography (TTE) and 3.5% with transesophageal echocardiography (TEE) (Table 3). With regards to means of transportation, 55% of IS and 78.8% of HS patients used the public emergency service to transfer to the hospital (Table 3). The vast majority of patients were transferred directly to the hospital (79.9% of IS and 77.4% of HS patients). For IS patients, prehospital time delay (time from stroke onset to emergency room) was 180 min (90–420). The median time from presentation at the emergency room (ER) to the first CT scan was 60 min (30–90) and the time delay from stroke onset to first CT scan was 255 min (150–536) (Table 3).

## 4. Discussion

The SUN4P registry is the first study providing data from a prospectively collected cohort of consecutive first-ever stroke patients from nine representative national hospitals across the country. It provides an overview on key prehospital features, primary stroke quality indicators and secondary performance measures of acute stroke care in national health system (NHS) in Greece.

Overall, 4.6% of our patients received thrombolysis in the 4.5-h time window, although 16.6% patients were eligible. The thrombolysis rate in our study is comparable to the rate reported in other registries [8,16,17]. Data from a survey across 44 European countries, including Greece, showed that the overall thrombolysis rate was 7.3% with considerable inter- and intra-country variability, with 13 countries reporting thrombolysis rates of 10% or more, with the highest reaching 20.6% in the Netherlands [6]. According to the European survey, Greece reported 1% of stroke patients treated with thrombolysis, which is much lower than 4.6% in our study [6]. This result should be also seen through the prism of the COVID-19 pandemic and the extreme derangement it caused in hospital pathways globally. Still, this progress in thrombolysis rates in Greece needs further optimization to reach future targets for acute stroke services, i.e., guaranteeing access to recanalization therapies to 95% of eligible patients, achievement of intravenous thrombolysis rates above 15%, treating more than 90% of all stroke patients in dedicated stroke units [18]. Mass public education on stroke, development of comprehensive stroke units/stroke centers, care of stroke patients by dedicated stroke teams and application of guidelines adherence programs in hospitals treating acute stroke patients would be essential to optimize acute stroke care in Greece.

The most frequent reason for not performing thrombolysis in our study was time delays. The median time from symptom onset to emergency room was 180 min for IS and 150 min for HS patients, which to a large degree reflects the hesitation of patients to visit hospitals during the pandemic as well as the saturation of prehospital transfer services by COVID-19 patients. According to a systematic review of 73 studies including patients worldwide [19], the median time between symptom onset and ER ranged between 3 and 4 h. These findings highlight the need for further mass public education to increase awareness and recognition of early warning signs and particularly the importance of seeking immediate medical care. For IS patients, the time from stroke onset to first CT scan was 255 min (150–536). Additionally, the median time from admission to first scan was 60 min (30–90), with only 53.2% of acute stroke patients receiving the first scan in less than 1 h after presenting at the ER. According to recent American guidelines, the recommended time from admission to first scan should be less than 20 min in >50% of rt-PA eligible patients [20]. Nevertheless, in our study median door-to -needle time for thrombolyzed patients was 60 min, which is in accordance with the median door-to-needle time of 70 min reported in European registries [18]. Our data emphasize the need to reduce in-hospital delays in the emergent evaluation of acute strokes.

The low rate of thrombolysis in our registry could also be partially attributed to the lack of comprehensive stroke units, staffed by dedicated stroke teams. Only 15.4% of acute IS patients were treated in an acute stroke unit and 20.7% in an ICU/HDU with the vast majority of 65% being admitted in internal medicine or neurology wards. According to a European survey this seems to be the standard of care in most Eastern and Southern European countries where a comprehensive network of stroke units is lacking [6]. In the same study, the number of acute stroke units per one million population has been significantly associated with the number of intravenous thrombolysis delivered per million population [6]. Notably, in a registry-based retrospective analysis from Germany, the intravenous thrombolysis rate varied in relation to service level from 44.0% in stroke centers to 13.1% in hospitals without a stroke unit [21]. On the other hand, data from an observational study using data from the Australian Stroke Clinical registry showed that although more than 75% of IS patients were admitted to stroke units, only 7.5% in rural and 12.7% in urban areas received thrombolysis. This was explained by the fact that even if the minimum criteria for stroke units are met, many services are unable to use their stroke unit’s full potential, because of a lack of specialists input, which in some areas was improved by using a telemedicine program [22]. Consequently, not only stroke unit facilities but also well-trained, dedicated stroke teams, which are available in just a few public hospitals in Greece, are essential for increasing thrombolysis rates. Implementation of telemedicine programs could also be helpful in improving thrombolysis rates in rural areas taking into account the country’s geography.

With regard to quality indicators for the prevention of in-hospital complications in our registry, dysphagia screening was low, ranging from 21.6% to 41.6% for IS and HS, respectively. Dysphagia screening is essential in patients with acute stroke, since swallowing difficulties are present in up to 67% of stroke patients and consist of a prerequisite for aspiration pneumonia which subsequently increases the risk of mortality [23]. Venous thromboembolism (VTE) prophylaxis with LMWH was started more often in IS compared to HS patients (65% vs. 39.4%). The latter, if left untreated, may lead to pulmonary embolism which accounts for nearly 10% of deaths after stroke [23]. In this context, both dysphagia screening and VTE prophylaxes have been endorsed by international major stroke quality improvement organizations to ensure optimization of provided healthcare quality [24,25]. In our registry, 65% of IS patients received VTE prophylaxis in a similar [26] or even higher [27] proportion than other large registries whereas dysphagia screening was underused as it has been previously documented, albeit to a greater extent in our cohort [14,26]. Potential explanations for this include the tendency to perform screening only in patients with severe strokes at high risk for aspiration, the absence of consensus regarding the most appropriate screening tool and the significant shortage of medical and nursing personnel during this period as many physicians and nurses were allocated in COVID-19-treating wards [23].

Numerous secondary prevention strategies have been found to be effective in reducing mortality rate after stroke [23]. For this purpose, these strategies have been implemented in daily clinical routine across countries of Europe [13] and the USA [26] to achieve the best possible quality of care and stroke recurrence reduction. In this direction, the initiation of secondary prevention measures such as antiplatelet therapy and anticoagulant therapy, lipid-lowering and blood pressure–lowering therapy, smoking cessation advice and carotid endarterectomy in eligible patients have been closely monitored in our registry. In our study, appropriate secondary prevention with antithrombotic, antihypertensive and lipid-lowering treatment was started in the vast majority of IS patients. Of note, all these strategies were generally utilized in a similar proportion to other large registries [14,26,27].

Assessment by a physiotherapist and early stroke rehabilitation is another performance measure aiming to improve functional outcomes in stroke patients [23]. In our registry, the proportion of patients assessed by a physiotherapist/occupational therapist was also remarkably lower compared to other registries [26,27,28]. Recovery from stroke depends on physical, occupational and speech therapy offered through organized rehabilitation programs. In our study, most stroke survivors (85% of IS and 66% of HS) were discharged home, without support from early discharge and community rehabilitation service (EDS). Only 12% of IS and 23% of HS patients were transferred to a comprehensive rehabilitation center, although it is known that up to 85% of stroke survivors have disabled motor function, almost 50% have impaired cognition and one third of patients have speech difficulties [18]. Notably, 34% of IS and 40% of HS patients in our study were discharged with severe disability (mRS ≥ 3) and almost 25% of IS and HS survivors remained dependent at 90 days. This reflects the limited availability of rehabilitation services for stroke survivors in Greece. Consequently, the majority of patients return to their homes without receiving rehabilitation [9] and just a minority of patients participated in rehabilitation programs, more often in private centers, where the cost is partly covered by social security funds. Importantly, the possible impact of the COVID-19 pandemic should be acknowledged as it prevented stroke patients from seeking standard rehabilitation therapy [29].

Brain parenchymal and vascular imaging constitutes an important quality indicator in the stroke care chain [23,30,31]. The vast majority of stroke patients in our study (94% of IS and 96.4% of HS) received brain imaging on admission. Computer tomography (CT) was the imaging modality of choice as first scan with magnetic resonance imaging (MRI) was used more often (in 36.8%) than CT (23.4%) as follow-up imaging in IS patients. Although MRI with diffusion weighted imaging (DWI) is more sensitive in the diagnosis of acute ischemic stroke and has also been proved as sensitive to acute intracranial hemorrhage, CT is a much easier and more available imaging method in the emergency departments. Thus MRI, which provides much more structural details, is preferred as follow-up examination, although its usefulness to guide treatment selection for the prevention of recurrent stroke has been questioned [20]. This is in accordance with the findings from other registries where the vast majority of IS patients received a CT scan as the first imaging modality [9,16,17,32].

Carotid imaging in strokes or TIA is currently performed in 70% to 80% of patients [23,32]. Imaging of the extracranial vessels in our study was offered to over half of the IS patients (53.4%), more often by cervical color-coded ultrasound (in 47.8%) and in fewer patients by CTA (11.3%), MRA (1.2%) or DSA (2.4%), whereas for intracranial imaging MRA (13.9%) was used more often. Early carotid imaging in IS patients is strongly recommended as carotid imaging is a necessary step to determine eligibility for carotid endarterectomy or stenting.

Our study has several strengths and limitations. Firstly, this is not a nationwide registry and therefore, results should not be extrapolated to the whole country. However, this is a prospective multicenter study across nine public hospitals in Greece, representative for the country’s geography, giving an overview of acute stroke care in Greece. Secondly, the COVID-19 pandemic has grossly influenced results because of in-hospital delays before the implementation of the COVID-19 code [33] and allocation of resources and of ICU/HDU beds to patients suffering from COVID-19. All participating hospitals were heavily involved in the management of COVID-19 patients, and many participating sites, especially the departments of Internal Medicine, had to operate under crucial shortages of medical and nursing personnel, as it was allocated to the COVID-19 wards. Additionally, there was a significant reduction in stroke admissions during the first months of the pandemic worldwide [34,35,36]. However, it should be stressed that even during the pandemic, all consecutive first-ever stroke patients admitted in contributing hospitals were registered in our study. In addition, a separate analysis comparing baseline data, stroke characteristics and mortality between the pre-COVID-19 era and COVID-19 era in the total population showed that there was no significant difference in stroke subtype, acute stroke severity, mean age, male gender proportion and in-hospital mortality (Supplementary Table S1). Finally, data on endovascular treatment with thrombectomy have not been presented as there were just a few cases (<10) in our registry. This is because thrombectomy services in most tertiary public hospitals in Greece were only recently organized, after the initiation of the study.

These initial results of the SUN4P registry represent an important step for the evaluation and improvement of the quality of acute stroke care in Greece. Although stroke care is being provided in a consistent manner for some performance measures, e.g., secondary prevention, heterogeneity in stroke care and treatment gaps were found in several other parameters like thrombolysis, admission to dedicated stroke units, screening for dysphagia, and referral to rehabilitation. Ongoing efforts to define performance measures and incorporate them into common national standards for stroke-related quality programs, could result in a national registry that will be able to identify, enable, and monitor improvements in hospital-based acute stroke care with substantial benefits to stroke patients and their families. Collecting common performance measures for stroke quality in a comprehensive way allows for meaningful comparisons of care quality between different health systems leading to the reduction of inequalities in acute stroke care between countries worldwide.

## Figures and Tables

**Table 1:** Stroke risk factors, comorbidities and classification of the study population.

Characteristic	Ischemic *n* = 755 (%)	Hemorrhagic * n = 137 (%)	*p* Value
Age (years) (mean, SD)	75.6 (13.6)	75.8 (13.2)	0.419
Men	368 (48.7)	79 (57.7)	0.063
NIHSS scale on admission (median, quartiles)	6 (3–11)	12 (5–23)	0.000
Pre-hospital mRS 0–1	591 (78.3)	113 (82.5)	0.306
Medical history			
Hypertension	522 (69.1)	94 (68.6)	0.920
Diabetes	208 (27.5)	22 (16.1)	0.004
Current smoking	184 (24.4)	27 (19.7)	0.275
Hyperlipidemia	288 (38.1)	40 (29.2)	0.054
Atrial Fibrillation	228 (30.2)	37 (27.0)	0.478
Coronary Artery Disease	101 (13.4)	16 (11.7)	0.680
Prosthetic cardiac valve diseases	20 (2.6)	3 (2.2)	1.000
Known carotid artery diseases	32 (4.2)	4 (2.9)	0.638
Heart failure	65 (8.6)	7 (5.1)	0.231
Symptomatic Peripheral Artery Disease	31 (4.1)	2 (1.5)	0.215
Previous TIAs	66 (8.8)	15 (10.9)	0.419
Heavy alcohol consumption	59 (7.8)	13 (9.5)	0.497
Active cancer	36 (4.8)	4 (2.9)	0.499
BMI (mean, SD)	27.6 (4.8)	27.8 (5.2)	0.919
TOAST classification of ischemic strokes			
Large vessel atherosclerotic	71 (9.4)		
Cardioembolic	240 (31.8)		
Lacunar	117 (15.5)		
Other	16 (2.1)		
Cryptogenic	311 (41.2)		
ESUS	47 (6.2)		
Multiple causes	9 (1.2)		
Incomplete investigation	255 (33.8)		

NIHSS, National Institute of Health Stroke Scale; mRS, modified Rankin Scale; TIAs, transient ischemic attack; BMI, Body Mass Index; ESUS, Embolic Stroke Undetermined Source. Non-continuous data are presented as percentages; * 13 cases were subarachnoid hemorrhage.

**Table 2:** Quality indicators in patients with acute ischemic and hemorrhagic stroke.

Characteristic	Ischemic *n* = 755	Hemorrhagic *n* = 137	*p* =
Outcomes at discharge			0.000
Dead	68 (9.0)	43 (31.4)	
Depended (mRS 3–5)	260 (34.4)	55 (40.1)	
Outcomes at 90 days			0.000
Dead	92 (12.2)	48 (35.0)	
Depended (mRS 3–5)	191 (25.3)	39 (28.5)	
Discharge destination (alive cases)	687	94	0.000
Patients or relatives’ home	587 (85.4)	62 (66.0)	
Rehabilitation center	83 (12.0)	22 (23.4)	
Nursing home	8 (1.2)	4 (4.3)	
Transferred to another hospital	9 (1.3)	6 (6.4)	
Coordination of care			
Patients treated in Acute Stroke Unit	116 (15.4)	11 (8.0)	0.024
Patients treated in ICU and HDU	156 (20.7)	46 (33.6)	0.001
Diagnosis			
Brain Imaging on admission (CT scan and or MRI)	712 (94.3)	131 (95.6)	0.684
Time from admission to 1st brain imaging (minutes, median, quartiles)	60 (31–105)	60 (30–90)	0.864
First brain imaging ≤60 min	379 (53.2)	73 (55.7)	0.350
Vascular imaging of carotid arteries	396 (53.4)	-	
Preservation of neural tissue			
Eligible for thrombolysis	125 (16.6)	-	
Thrombolytic therapy	35 (4.6)	-	
Door-to-needle time in 35 cases that received thrombolysis (minutes)	60 (41–90)	-	
Prevention of complications			
Screening for dysphagia	163 (21.6)	57 (41.6)	0.000
Venous thromboembolism prophylaxis *	491 (65.0)	54 (39.4)	0.000
Restoration of function			
Assessment by physiotherapist and occupational therapist	225 (29.8)	46 (33.6)	0.419
Initiation of secondary prevention			
Cardiac arrhythmia detection	230 (30.5)	-	
Antiplatelet therapy inhospital	653 (86.5)	-	
Antiplatelet therapy on discharge in non-AF cases (535 surviving patients)	505 (94.4)	-	
Anticoagulant therapy in patients with AF on discharge (153 patients)	112 (73.2)	-	
Lipid-lowering therapy (survived)	480/688 (69.8)	31/94 (33.0)	0.000
Blood pressure–lowering therapy (survived)	424/688 (61.6)	65/94 (67.1)	0.174
Smoking cessation advice (survived current smokers)	135/173 (78.0)	14/22 (63.6)	0.180
Carotid endarterectomy within 14 days in 26 eligible patients	6/26 (23.1)	-	
Outcomes (90 days)			
Dead	137 (18.1)	52 (38.0)	0.000
Depended (mRS 3–5)	206 (27.3)	43 (31.4)	0.352

mRS, modified Rankin Scale; AF, Atrial Fibrillation, ICU, Intensive Care Unit, HDU, High-dependency Unit; * Low Molecular Weight heparin use (low dose). For patients with ICH after 2 days from admission. Continuous data are presented as median (quartiles) and non-continuous data as percentages %.

**Table 3:** Secondary performance measures.

Variable	Ischemic *n* = 755	Hemorrhagic *n* = 137	*p* Value
Length of hospital stay (days)	6 (4–9)	9 (6–15)	0.000
Means of transportation			0.000
Public services	415 (55.0)	108 (78.8)	
Private	340 (45.0)	29 (21.2)	
Mode of transportation			0.301
Directly to the hospital	603 (79.9)	106 (77.4)	
From Health Centers	75 (9.9)	16 (11.7)	
From other hospital	64 (8.5)	15 (10.9)	
Inhospital stroke cases	13 (1.7)	0 (0.0)	
Patient delays (minutes)			
Stroke onset to emergency room	180 (90–420)	150 (75–348)	0.373
Emergency room to first CT scan	60 (30–90)	51 (30–90)	0.373
Stroke onset to 1st CT scan	255 (150–536)	210 (120–555)	0.864
Inhospital investigations			
First cerebral imaging <24 h	714 (94.6)	132 (96.4)	0.528
CT scan first	755 (100.0)	132 (100.0)	1.000
CT scan more than one	177 (23.4)	52 (38.0)	0.001
MRI study	278 (36.8)	31 (22.6)	0.001
Neck ultrasound	361 (47.8)	10 (7.3)	0.000
Cerebral Angiography ^1^	165 (21.9)	22 (16.1)	0.139
MRA extracranial arteries	11 (1.2)	0 (0.0)	0.389
MRA intracranial arteries	105 (13.9)	14 (10.2)	0.276
CT Angiography	85 (11.3)	13 (9.5)	0.656
DSA Angiography	18 (2.4)	2 (1.5)	0.755
Transthoracic echocardiography	298 (39.5)	21 (15.3)	0.000
Transesophageal echocardiography	26 (3.5)	1 (0.7)	0.105

Continuous data are presented as median (quartiles) and non-continuous data as percentages. ^1^ 51 cases had more than one angiography study.

## Data Availability

The original contributions presented in the study are included in the article/Supplementary Materials, further inquiries can be directed to the corresponding author.

## Supplementary Materials

The following are available online at https://www.mdpi.com/article/10.3390/jcm13030917/s1, Figure S1: Study design; Figure S2: Sites of participated study centers; Table S1: Comparison of baseline characteristics, stroke severity and mortality during COVID pandemic and outside COVID era.
