# Supplementary material for: Quality Indicators and Clinical Outcomes of Acute Stroke: Results from a Prospective Multicenter Registry in Greece (SUN4P)
Source: J Clin Med. 2024 Feb 5;13(3):917. doi: 10.3390/jcm13030917 (PMC10856279; doi:10.3390/jcm13030917)
Supplement: Supplementary file 1 [file jcm-13-00917-s001.zip › jcm-2789959-supplementary.pdf]

Supplementary material

Supplementary Table S1
Supplementary Figure S1
Supplementary Figure S2

Table S1. Comparison of baseline characteristics, stroke severity and mortality during Covid pandemic and outside Covid era.

	**Pre-Covid N=279**	**Covid period N=613**	*p*=
Mean age	74.4 (SD 14.0)	76.1 (13.3)	0.073
NIHSS	8.5 (SD 7.6)	9.4 (SD 8.2)	0.096
Male gender	51.6%	49.4%	0.564
Ischemic stroke	83.5%	85.2%	0.548
Inhospital Mortality	10.0%	13.5%	0.156

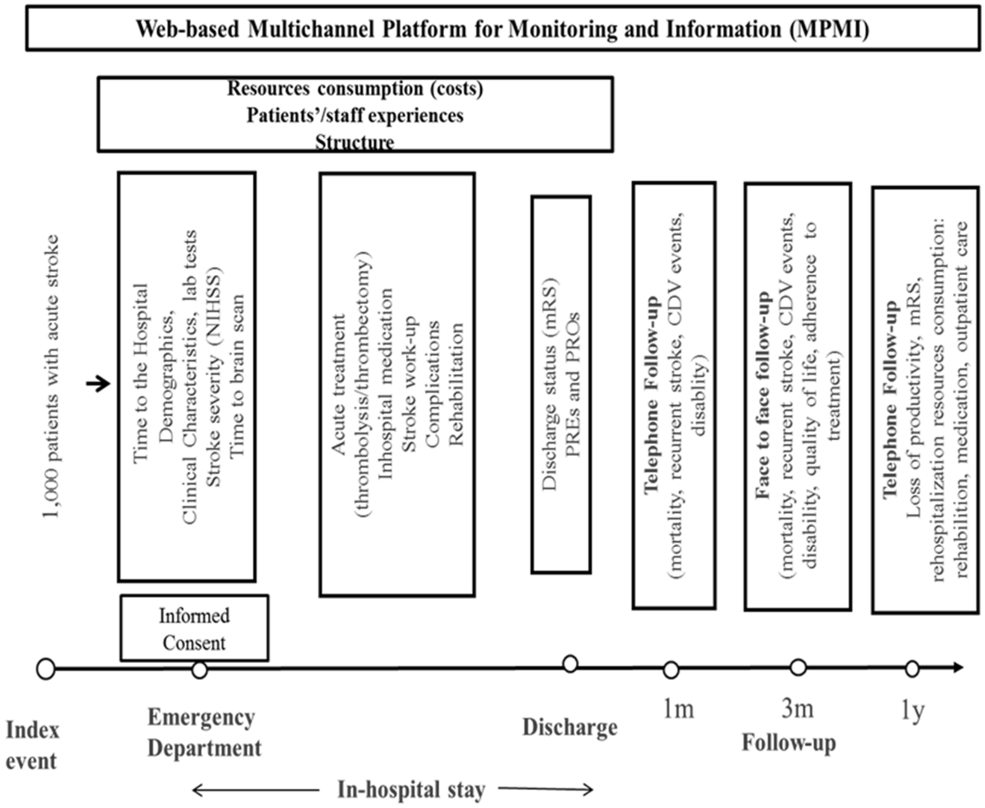

Figure S1. Study design.

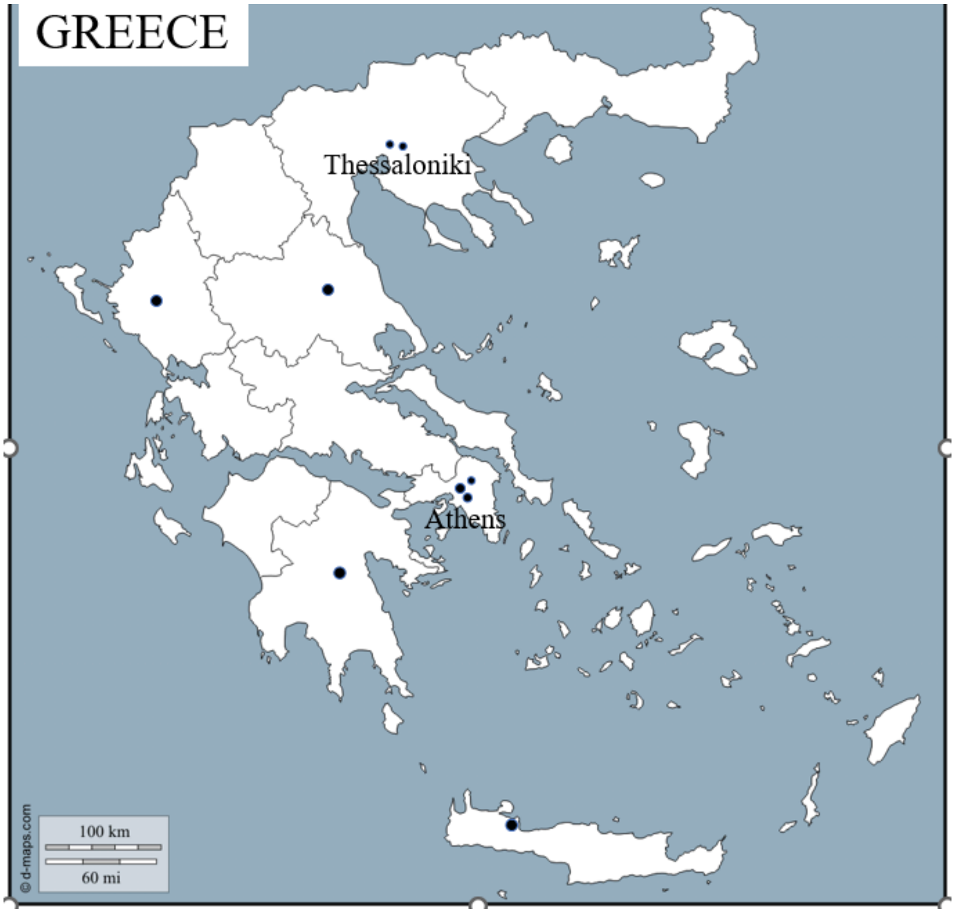

Figure S2. Sites of participated study centers.
